# Retraction: Corrigendum: Do Chinese children need parental supervision to manage their out-of-school visual art activities and academic work time?

**DOI:** 10.3389/fpsyg.2025.1595133

**Published:** 2025-03-27

**Authors:** Frontiers Editorial Office

The journal retracts the November 24 2022 article cited above.

Following publication, the authors contacted the Editorial Office to request the retraction of the cited article, stating that the article's conclusions and assertions were not sufficiently supported by their study data, which was found unreliable after an investigation by the authors' institution.

Following COPE recommendations, the authors were asked to provide the study's raw data for verification. However, the authors have remained unresponsive to multiple requests for the raw data for this study. Given extant concerns over the validity of the study, the article has been retracted.

This retraction was approved by the Chief Editors of Educational Psychology and the Chief Executive Editor of Frontiers.

The authors received a communication regarding the retraction and had a chance to respond. This communication has been recorded by the publisher.

